# Cr(VI) removal performance from wastewater by *microflora* isolated from tannery effluents in a semi-arid environment: a SEM, EDX, FTIR and zeta potential study

**DOI:** 10.3389/fmicb.2024.1423741

**Published:** 2024-07-01

**Authors:** Aké Henri Joël Aké, Nabil Rochdi, Martin Jemo, Mohamed Hafidi, Yedir Ouhdouch, Loubna El Fels

**Affiliations:** ^1^Laboratory of Microbial Biotechnologies, Agrosciences and Environment, Labeled Research Unit-CNRST N°4, Faculty of Sciences Semlalia, Universiry Cadi Ayyad, Marrakesh, Morocco; ^2^Laboratory of Innovative Materials, Energy and Sustainable Development (IMED-Lab), Cadi Ayyad University, Marrakesh, Morocco; ^3^Department of Physics, Faculty of Sciences Semlalia, Cadi Ayyad University, Marrakesh, Morocco; ^4^AgroBiosciences Program, College of Agriculture and Environmental Sciences, University Mohammed VI Polytechnic (UM6P), Ben Guerir, Morocco; ^5^African Sustainable Agriculture Research Institute (ASARI), College of Agriculture and Environmental Sciences, University Mohammed VI Polytechnic (UM6P), Laâyoune, Morocco

**Keywords:** chromium bioremediation, scanning electron microscopy, energy-dispersive X-ray spectroscopy, Fourier transform infrared spectroscopy, zeta potential measurements

## Abstract

Hexavalent chromium removal from the environment remains a crucial worldwide challenge. To address this issue, microbiological approaches are amongst the straightforward strategies that rely mainly on the bacteria's and fungi's survival mechanisms upon exposure to toxic metals, such as reduction, efflux system, uptake, and biosorption. In this work, scanning electron microscopy, energy-dispersive X-ray spectrophotometry, Fourier transform infrared spectroscopy, and zeta potential measurements were used to investigate the ability of chromium adsorption by *Bacillus licheniformis, Bacillus megaterium, Byssochlamys* sp., and *Candida maltosa* strains isolated from tannery wastewater. Scanning electron microscopy combined with energy dispersive X-ray spectroscopy revealed alterations in the cells treated with hexavalent chromium. When exposed to 50 mg/L Cr^6+^, *Bacillus licheniformis* and *Candida maltosa* cells become rough, extracellular secretions are reduced in *Bacillus megaterium*, and *Byssochlamys* sp. cells are tightly bound and exhibit the greatest Cr weight percentage. In-depth analysis of Fourier transform infrared spectra of control and Cr-treated cells unveiled Cr-microbial interactions involving proteins, lipids, amino acids, and carbohydrates. These findings were supported by zeta potential measurements highlighting significant variations in charge after treatment with Cr(VI) with an adsorption limit of 100 mg/L Cr^6+^ for all the strains. *Byssochlamys* sp. showed the best performance in Cr adsorption, making it the most promising candidate for treating Cr-laden wastewater.

## 1 Introduction

Chromium (Cr) and its salts are involved in plenty of industrial processes, such as the manufacturing of paints, dyes, plastics, and stainless steel, the wood treatment, and the leather tanning. Unlike its trivalent form (Cr^3+^), hexavalent chromium (Cr^6+^) poses a serious environmental problem (Fu et al., 2021; Lara et al., 2021; Wang et al., 2022; Aguilar et al., 2023; Mohanty et al., 2023) and triggers harmful effects that make its elimination essential (Wang et al., 2021; Aké et al., 2022; Alharbi et al., 2022). Moreover, Cr^6+^ is found to be particularly hazardous to health (Tangahu et al., 2020; Aparicio et al., 2021) and responsible for dysfunction in living organisms (Tripathi et al., 2018; Chromikova et al., 2022). Hence, Cr^6+^ removal has become the focus of attention in health and safety projects (Tumolo et al., 2020; Anupong et al., 2022; Ariram et al., 2022).

Based on these considerations, it is obvious that substantial efforts were deployed toward building environmentally friendly solutions for the treatment of chromium (VI). In this context, researchers developed biological methods making use plants and microorganisms to detoxify the environment (Xia et al., 2019; Yasir et al., 2021). Bacteria and fungi repeatedly demonstrated their ability to resist or remove Cr^6+^ present in environmental or culture media (Chang et al., 2016; Kalsoom K. et al., 2021; Aké et al., 2023). Indeed, it has been shown that anaerobically or aerobically cultivated bacteria in laboratory can eliminate the synthetic Cr^6+^ contained in culture media via a number of mechanisms (Lin et al., 2020; Plestenjak et al., 2022). In an earlier work, we demonstrated the aerobic reduction of Cr^6+^ to trivalent chromium by cultures of *Bacillus licheniformis, Bacillus megaterium, Byssochlamys sp*., and *Candida maltosa* strains isolated from tannery effluents (Aké et al., 2023). Similar reduction of Cr^6+^ to Cr^3+^ was observed for *Penicillium* sp. PL17, *Fusarium proliferatum* FBL1, *Aspergillus fumigatus* ML43, and *Rhizopus* sp. CUC23 (Bibi et al., 2018). The ability to reduce Cr^6+^ is one of the mechanisms of strain resistance to Cr (Aké et al., 2023) that include biosorption, bioaccumulation, biotransformation, and efflux systems (Gang et al., 2019; Xia et al., 2019; Ayele et al., 2021; Elahi et al., 2022; Plestenjak et al., 2022). Microorganisms can act as adsorbents, similar to carbon nanotubes, activated carbon, graphene oxide, polymers, clays, green slow-releasing denaturing colloidal substrates (such as gelatin, agar, and cane molasses), and their recently-used modifications (Sathvika et al., 2019). Once Cr^6+^ is in the vicinity of the cell, it can be attached (adsorption) to the cell's surface by specific molecules (COOH, ${\text{NH}}_{3}^{+}$, etc.) and subsequently reduced to Cr^3+^. This reduction may be spontaneous or due to a cytochrome network in the cell wall (Thatoi et al., 2014; Upadhyay et al., 2017; Li et al., 2021; Mat Arisah et al., 2021). Chromium adsorption can be checked by measuring its content after the cell washing (exposure), allowing one to quantitatively assess the concentrations of adsorbed ionic species, especially Cr^6+^ and Cr^3+^ ions. On the other hand, the researchers can identify the families of molecules involved in the adsorption of Cr^6+^ using Fourier transform infrared spectroscopy (FTIR) analyses (Kaduková and Virčíková, 2005; Sharma et al., 2022). Besides adsorption on the cell surface, chromium can penetrate the microbial cell through sulfate and phosphate transporters (${\text{SO}}_{4}^{2-}$, ${\text{PO}}_{4}^{3-}$), and Cr^6+^ can undergo enzymatic or non-enzymatic reduction (indirect reduction) inside the cell (Thatoi et al., 2014). The ability of microorganisms to adsorb Cr^6+^ and Cr^3+^ was also demonstrated using scanning electron microscopy (SEM) combined to energy dispersive X-ray spectroscopy (EDX) (Abo-Alkasem et al., 2022; Chromikova et al., 2022). At high concentrations, the authors found cellular modifications due to high Cr^6+^ concentrations (Abo-Alkasem et al., 2022; Chromikova et al., 2022). Therefore, the combination of FTIR and SEM-EDX analyses is a powerful tool to understand the Cr resistance mechanisms. While FTIR relies on the vibrational behavior of Cr-loaded cells to identify the chemical environments and involved functional groups, SEM and EDX provide an overview of the surface morphology and elemental contents in treated specimens. However, this approach is not sufficient since it does not provide insight into the electrical phenomena that occur between Cr molecules and ligands on the surface of microorganisms. The study of the electrical charges surrounding the cells enables a better understanding of the interactions between metals and microbial cells. To that end, zetametry is an effective tool to assess the electric charge that a particle, in suspension or in solution, acquires from the surrounding ion cloud using the potential difference between the particle-medium interfacial layer and the medium (the so-called zeta potential) (Samaké, 2008; Al-Jubory et al., 2020; Youssef et al., 2020). The combination of FTIR, SEM, EDX, and zetametry measurements has been used successfully in the study of Cd(II) biosorption by *Bacillus cereus* RC−1 cells, for instance (Huang et al., 2013) but not in a study of Cr^6+^ adsorption by bacteria and fungi.

Accordingly, the aim of the present work is to examine the potential of chromium (VI) adsorption and its biotransformation into chromium (III) using indigenous strains of *Bacillus licheniformis, Bacillus megaterium, Byssochlamys* sp., and *Candida maltosa* that were isolated from tannery effluents. SEM-EDX characterizations were carried out for the investigated biomasses to probe the modifications in morphology and chemical makeup after Cr loading. Similarly, FTIR characterizations were also performed to reveal the major vibrational alterations after treatment with chromium in order to incriminate the possible functional groups involved in the metal-microbial strain interactions. In this respect, we carried out in-depth analyses of vibrational modes in the investigated strains to provide a useful literature impetus about the infrared signature of investigated strains. Moreover, zeta potential characterizations were performed to assess the modifications in the electrical behavior of the strain environment upon Cr loading.

## 2 Materials and methods

### 2.1 Microorganism isolation

The raw tannery effluent was sampled from Bab Dbagh and the industrial district at Marrakech tannery manufacturing, which operates with chrome-tanning processes. *Bacillus licheniformis, Bacillus megaterium, Byssochlamys* sp., and *Candida maltosa* strains were isolated from tanneries effluents and identified using the previously reported molecular methods (Aké et al., 2023).

### 2.2 SEM-EDX analysis

Culture pellets were subjected to SEM and EDX characterizations in order to confirm Cr adsorption and to examine the impact of Cr on cell morphologies. The experimental procedure was similar to that applied by Karthik et al. (2017). After incubation for 96 h, the cells in 100-μL precultures with 0, 5, and 50 mg/L Cr^6+^ were harvested by centrifugation at 5000 rpm. After washing with a 0.1 mM phosphate buffer solution (pH 7) to remove the cells from the culture medium, the pellets were fixed overnight in 3% glutaraldehyde at 4 °C. Subsequent dehydration cycles using ethanol at different volume concentrations (20%, 40%, 60%, 80%, 90%, and 100%) were applied to the pellets treated with glutaraldehyde. The surface morphology and the chemical composition of dehydrated strains supported on carbon sheets were analyzed using a TESCAN VEGA 3 scanning electron microscope equipped with an energy-dispersive X-ray spectrometer. The SEM images were obtained by detecting the secondary electrons emitted by the samples when excited with a beam of 10 keV energy. The EDX spectra were recorded at a beam energy of 5 keV to detect the prominent Cr Lα radiation with an energy of about 0.57 keV. Further analyses were performed with a beam energy of 10 keV to check the detection of Cr Kα and Kβ characteristic lines located at 5.41 and 5.95 keV, respectively. Microbial cells cultured without Cr^6+^ were used as a control and are referred to as “native” hereafter.

### 2.3 FTIR analysis

In order to determine the impact of Cr^6+^ on the functional groups of the microbial cell surface, 100 μL of preculture (24 h) of each strain was introduced into Luria-Bertani (LB) broth containing 50 mg/L Cr^6+^ (30 °C, pH 7). At the same time, a control without Cr^6+^ was prepared for each strain. After 96 h incubation, the cells were then collected in 15 mL tubes and centrifuged at 5000 rpm (20 min, 4 °C). After that, the preparation procedure was performed according to the analytical method used by Maurya et al. (2022). The pellets were washed with a 0.1 mM phosphate buffer solution (pH 7) to remove to remove the cells from the culture medium, then freeze-dried using a Martin Christ Alpha 1-2 LO plus lyophilizer. A total 0.099 g of KBr powder was mixed with 0.001 g of each powder pellet obtained and grinded in a mortar. After mixing, the product was ground, homogenized, and then pressed into pellets using a compressor. The as-obtained pellets were introduced into a Bruker Vertex 70 FTIR spectrometer. Each IR spectrum was obtained by averaging 31 scans recorded in the 400–4000 cm^−1^ range with a wavenumber step of 2 cm^−1^.

### 2.4 Zeta potential analysis

In order to understand the electrostatic interactions between the microbial cell and Cr^6+^, the zeta potential measurements were carried out using a Malvern zetasizer ver. 7.12. To that end, a modified experiment derived from Beiranvand et al. (2022) was applied. Different concentrations of Cr^6+^, namely 25, 50, 100, 250, 500, and 800 mg/L, were prepared in distilled water. The inoculum of each strain was added to each Cr^6+^ concentration (5 mL). Positive controls, i.e., solutions without inocula, were also prepared. The zeta potential was also measured in pure distilled water.

### 2.5 Statistical analysis

The experiments from the study were carried out in triplicate. The data obtained were statistically analyzed and are presented with the appropriate standard deviation. ANOVA tests were carried out to better understand the effect of the microbial strain treatments. *Post-hoc* tests were then applied to observe the significance between groups. The least significant differences among means were evaluated at the 5% significance level. IBM SPSS Statistics 25 was used for the statistical analysis of the data.

## 3 Results

### 3.1 SEM analysis

**Figure 1:**
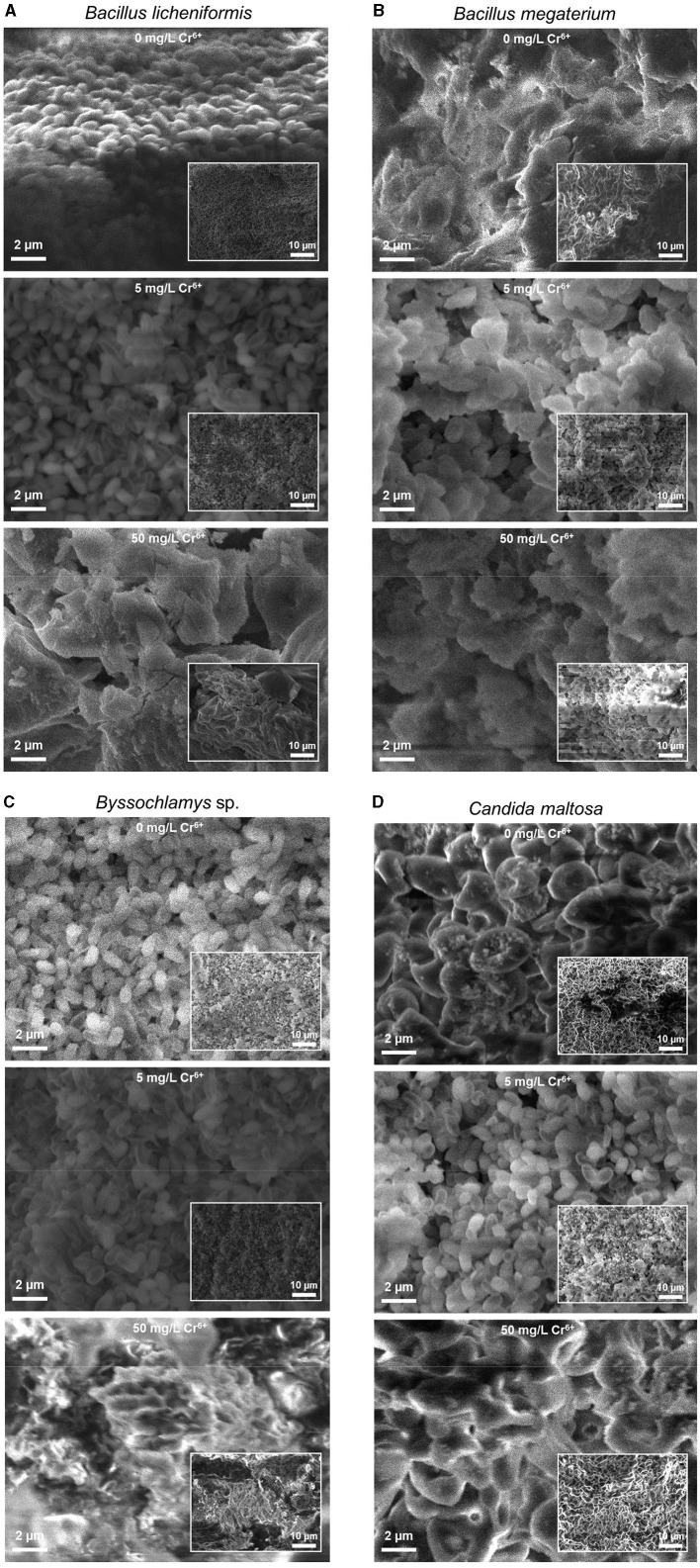
Secondary electron SEM images of the native (upper images) and treated strains with 5 (middle images) and 50 mg/L Cr^6+^ (bottom images) in LB medium for *Bacillus licheniformis* **(A)**, *Bacillus megaterium* **(B)**, *Byssochlamys* sp. **(C)**, and *Candida maltosa* **(D)** strains isolated from tannery effluents. The SEM images and the inset low-magnification secondary electron SEM images are displayed with the same scales for all the samples.

The SEM images of Figure 1 depict the morphologies of the *Bacillus licheniformis, Bacillus megaterium, Byssochlamys* sp., and *Candida maltosa* strains without Cr treatment (upper images) and after exposure to 5 and 50 mg/L of hexavalent chromium (middle and bottom images, respectively). These SEM images are displayed with the same magnifications to show specific features in structure of each sample.

In the case of the Cr^6+^-free *Bacillus licheniformis* strains, the cells are smooth, compact, and firmly grouped in the form of asymmetric clusters (Figure 1A). In addition, the native cells are typically regular in shape, and their assembly gives rise to a domed structure with corrugations. After contact with 5 mg/L Cr^6+^, the cells are still smooth and clustered, with a quite regular cell size. In other words, both of the native bacterial biomass and that exposed to 5 mg/L Cr^6+^ show similar cell surfaces. However, at 50 mg/L Cr^6+^, the appearance of the bacterial biomass is altered and shows wrinkled, rough, and irregular clusters of less distinct cells, with possible extracellular secretions.

In contrast, the *Bacillus megaterium* strains do not show very distinct cell shapes, either in the absence or presence of Cr^6+^ (Figure 1B). The cells form aggregates with apparent depressions and exhibit a very compact texture with irregular edges that appear to be bound with an extracellular matrix. The visual examination of SEM images suggests a higher content of extracellular secretions in the control than in the strains grown at 5 and 50 mg/L Cr^6+^.

For the *Byssochlamys* sp. strains (Figure 1C), the control and the strain loaded with 5 mg/L Cr^6+^ show the same appearance. Indeed, the cells appear smooth, less bound, and exhibit a few corrugations. In contrast, the strain cultured with 50 mg/L Cr^6+^ show a different topography since the cells are tightly bound and offer a compact space upon exposure to Cr^6+^.

Similarly, the *Candida maltosa* control cells and those treated with 5 mg/L Cr^6+^ show a smooth outline morphology with visible depression regions (Figure 1D). The SEM image of native strains shows structures of a regular size in the 1–2 μm range, which corresponds to the typical size of the *Candida maltosa* cells. However, at 5 mg/L Cr^6+^, some cells are less visible and form irregular blocks. At a concentration of 50 mg/L Cr^6+^, several cells had lose their initial structures and appear slightly rough and deformed.

### 3.2 EDX analysis

**Figure 2:**
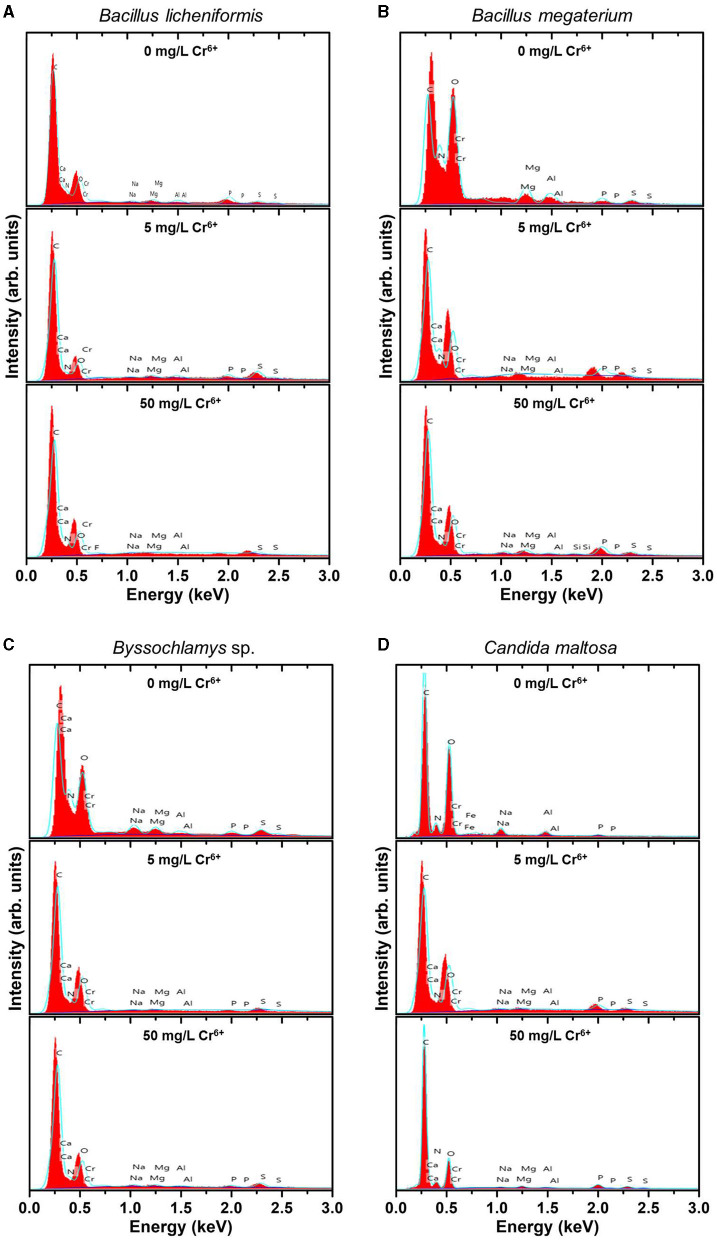
EDX spectra of the native (upper spectra) and treated strains with 5 mg/L Cr^6+^ (middle spectra) and 50 mg/L Cr^6+^ (bottom spectra) in LB medium for *Bacillus licheniformis* **(A)** *Bacillus megaterium* **(B)**, *Byssochlamys* sp. **(C)**, and *Candida maltosa* **(D)** strains isolated from tannery effluents.

EDX analysis was performed to determine whether the microbial cell surfaces are capable of bio-adsorbing Cr. Figure 2 shows the EDX analysis of *Bacillus licheniformis, Bacillus megaterium, Byssochlamys* sp., and *Candida maltosa* strains grown without Cr treatment and after loading with 5 and 50 mg/L Cr^6+^. The determined weight and atomic percentages of Cr (Cr wt.% and Cr at.%, respectively) are given in Table 1.

**Table 1:** Weight and atomic percentages of *Bacillus licheniformis, Bacillus megaterium, Byssochlamys* sp., and *Candida maltosa* microbial biomasses before (0 mg/L Cr^6+^) and after treatment with 5 and 50 mg/L Cr^6+^.

**Strain**	**Concentration (mg/L)**	**Cr contents**	
		**(wt.%)**	**(at.%)**
*Bacillus licheniformis*	0	0.00	0.00
	5	1.10	0.30
	50	0.29	0.08
*Bacillus megaterium*	0	0.00	0.00
	5	0.52	0.14
	50	0.25	0.07
*Byssochlamys* sp.	0	0.00	0.00
	5	0.51	0.13
	50	0.35	0.09
*Candida maltosa*	0	0.00	0.00
	5	0.62	0.17
	50	0.23	0.06

Besides the characteristic C K line (overlapping of the Kα and Kβ peaks) located at about 0.28 keV, the EDX spectra of all the control strains show no signature of the Cr Lα feature (at 0.57 keV) but the neighboring O K feature arising from the overlapping Kα and Kβ peaks (located at ~0.52 and 0.53 keV, respectively). The nitrogen signature (expected at 0.39 and 0.4 keV) is not discernible from the prominent carbon peak. Additional small peaks at energies consistent with the Kα and Kβ lines of Na (1.04 and 1.07 keV), Mg (1.25 and 1.3 keV), Al (1.49 and 1.55 keV), P (2.01 and 2.14 keV), and S (2.31 and 2.47 keV) are also observed. The presence of these peaks probably originates from compounds present in the cell wall.

In contrast, the biomasses that has grown in the Cr^6+^-amended culture media show the abovementioned Cr peak (Cr Lα line at 0.57 keV). After treatment of the microorganisms with 5 mg/L Cr^6+^, the EDX analyses reveal Cr weight percentages of 1.1, 0.52, 0.51, and 0.62 and atomic percentages of 0.30, 0.14, 0.13, and 0.17 for *Bacillus licheniformis, Bacillus megaterium, Byssochlamys* sp., and *Candida maltosa*, respectively. Nevertheless, chromium contents decrease in each strain after exposure to 50 mg/L Cr^6+^; the Cr weight percentages were 0.29, 0.25, 0.35, and 0.23, and the Cr atomic percentages were 0.08, 0.07, 0.09, and 0.06 for *Bacillus licheniformis, Bacillus megaterium, Byssochlamys* sp., and *Candida maltosa*, respectively.

### 3.3 FTIR analysis

The FTIR spectra of the investigated strains consist of numerous overlapping bands, most of which are derived from protein, lipid, amino acid, and carbohydrate functional groups. Table 2 summarizes the prominent identified vibrations and corresponding assignments for the *Bacillus licheniformis, Bacillus megaterium, Byssochlamys* sp., and *Candida maltosa* specimens, before and after chromium loading.

**Table 2:** Frequencies^a^ and tentative assignment of prominent vibrational modes^b^ in native and chromium(VI)-treated *Bacillus licheniformis, Bacillus megaterium, Byssochlamys sp*., and *Candida maltosa* strains as identified from FTIR measurements.

***B. licheniformis***		***B. megaterium***		***Byssochlamys sp***.		***C. maltosa***		**Assigned vibrations**	**Comments**
**Native**	**Treated**	**Native**	**Treated**	**Native**	**Treated**	**Native**	**Treated**		
3761	3761	3755	3759	3755	3759	3756	3758	Water ν_as_(O–H)	
–	–	3438 3435^*^	–	–	–	–	–	Carbohydrate ν(O–H)	
3302 3282^*^	3299 3282^*^	– 3274^*^	3299 3284^*^	3297 3279^*^	3298 3281^*^	3304 –	3304 –	Protein / peptide amide A ν(N–H)	Overlapped with hydroxyl group ν(O–H) and amino acid ν_as_(–${\text{NH}}_{3}^{+}$)
– 3067^*^	– 3066^*^	– 3066^*^	3093 3067^*^	3094 3068^*^	– 3067^*^	– 3061^*^	– 3064^*^	First overtone of the amide II band	Also consistent with a polyglycine ν(N–H) band
2962 2964^*^	2963 2964^*^	2966 2963^*^	2961 2964^*^	2963 2965^*^	2962 2964^*^	2958 2965^*^	2958 2964^*^	Lipid / fatty acids –CH_3_ ν_as_(C–H)	
2929 2926^*^	2932 2928^*^	2933 2925^*^	2930 2926^*^	2934 2928^*^	2931 2927^*^	2928 2927^*^	2927 2925^*^	Lipid / polyglycine >CH_2_ ν_as_(C–H)	
– 2876^*^	– 2874^*^	– 2874^*^	– 2876^*^	2883 2874^*^	– 2875^*^	– 2877^*^	– 2877^*^	Lipid –CH_3_ ν_s_(C–H)	
2852^*^	2853^*^	2852^*^	2853^*^	2853^*^	2853^*^	2852^*^	2852^*^	Lipid / polyglycine >CH_2_ ν_s_(C–H)	
2375 2372^*^	2374 2372^*^	2373 2371^*^	2374 2371^*^	2373 2372^*^	2373 2371^*^	2372 2369^*^	2373 2370^*^	Physisorbed CO_2_ ν_as_(CO_2_)	Most likely a spurious band due to contamination with CO_2_
2340 2341^*^	2339 2342^*^	2340 2342^*^	2340 2341^*^	2340 2341^*^	2341 2342^*^	2341 2340^*^	2341 2342^*^	Gaseous CO_2_ ν_as_(CO_2_)	Most likely a spurious band due to contamination with CO_2_
–	–	2120	2128	2118	2129	2120	2124	Amino acid ν_as_(–${\text{NH}}_{3}^{+}$)	Probably overlapped with Water sc(H_2_O) and ρ(H_2_O)
1749^*^	1747^*^	–	1746^*^	1746^*^	1744^*^	1747^*^	1748^*^	Lipid / amino acid ν(C=O)	
1686^*^	1689^*^	1684^*^	1689^*^	1693^*^	1690^*^	1697^*^	1691^*^	Polyglycine ν(${\text{CO}}_{2}^{-}$)	Also consistent with β-antiparallel pleated sheet protein / peptide amide I ν(C=O)
1656 1662^*^	1658 1662^*^	– 1660^*^	1657 1662^*^	1658 1662^*^	1656 1662^*^	1655 –	1657 1662^*^	α-helix protein / peptide amide I ν(C=O)	With smaller ν(C–N) and δ(N–H) contributions Probably overlapped with ester ν(C=C)
– 1636^*^	– 1635^*^	1641 1632^*^	– 1635^*^	–	1636^*^	–	–	Amino acid / polyglycine μ_as_(${\text{NH}}_{3}^{+}$)	Also consistent with β-sheet protein / peptide amide I ν(C=O) and water δ(O–H)
–	–	–	–	1617^*^	–	1621^*^	1626^*^	Amino-/fatty acids ν_as_(${\text{CO}}_{2}^{-}$) and μ_as_(–${\text{NH}}_{3}^{+}$)	
1573^*^	1572^*^	1568^*^	1574^*^	1573^*^	1574^*^	1572^*^	1572^*^	Polyglycine μ_as_(${\text{NH}}_{3}^{+}$)	
1546 1544^*^	1544 1544^*^	1550 1546^*^	1546 1544^*^	1546 1544^*^	1546 1544^*^	1545 1544^*^	1545 1545^*^	α-helix protein / peptide amide II δ(N–H) and ν(C–N) Amino-/fatty acids ν_as_(${\text{CO}}_{2}^{-}$)	Also consistent with amino acid μ(NH_2_)
1515^*^	1515^*^	1519^*^	1514^*^	1511^*^	1515^*^	1512^*^	1514^*^	Amino acid tyrosine ring vibration and μ_s_(–${\text{NH}}_{3}^{+}$)	Also consistent with polyglycine μ(N–H)
1463^*^	1463^*^	1463^*^	1463^*^	1461^*^	1464^*^	1464^*^	1465^*^	Lipid δ_as_(CH_3_) and sc(CH_2_)	
1444 1442^*^	1444 1442^*^	1446 1444^*^	1445 1443^*^	1444 1442^*^	1444 1442^*^	1447 1443^*^	1444 1443^*^	Lipid cyclohexyl (fatty acids) μ(CH_2_)	
–	–	1400 1404^*^	– 1401^*^	–	–	1398^*^	1401	Amino/fatty acids / Polyglycine ν_s_(${\text{CO}}_{2}^{-}$)	
1386 1380^*^	1386 1378^*^	– 1380^*^	1386 1378^*^	1385 1376^*^	1386 1377^*^	1387 1376^*^	1385 1377^*^	Lipid δ_s_(CH_3_)	Also consistent with Carbohydrate μ(CH)
1315 1314^*^	1314 1315^*^	1317 1320^*^	1316 1314^*^	1314 1314^*^	1315 1314^*^	1312 1313^*^	1314 1314^*^	Polysaccharide μ(OH)	Also consistent with amine τ(CH_2_)
1275 1279^*^	– 1280^*^	1280 1280^*^	1278 1280^*^	1276 1279^*^	1277 1280^*^	– 1280^*^	– 1280^*^	α-helix protein amide III ν(C–N) and δ(N–H)	With smaller ν(C=O) and δ(O=C–N) contributions
1242 1240^*^	1243 1237^*^	1245 1241^*^	1239 1240^*^	1243 1240^*^	1241 1238^*^	1242 1235^*^	1244 1237^*^	Phospholipid / phosphodiester >${\text{PO}}_{2}^{-}$ν_as_(P=O)	
1156 1163^*^	– 1169^*^	1151 1160^*^	1159 1165^*^	1163 1165^*^	1160 1166^*^	– 1161^*^	– 1159^*^	Polysaccharide glycosidic link ν(C–O–C) Lipid ν_as_(CO–O–C)	Also consistent with saccharide ν(C–C), secondary alcohol ν(C–O), and amino acid ρ(${\text{NH}}_{3}^{+}$)
1110 1117^*^	1113 1115^*^	– 1120^*^	– 1118^*^	1111 1118^*^	1108 1118^*^	– 1118^*^	– 1111^*^	Phospholipid / phosphodiester ν_s_(${\text{PO}}_{2}^{-}$)	Also consistent with saccharide secondary alcohol ν(C–O)
1069 1069^*^	1075 1068^*^	1071 1078^*^	1068 1065^*^	1069 1072^*^	1071 1067^*^	1073 1079^*^	1072 1077^*^	Polysaccharide ring ν(C–O) and ν(C–C) ${\text{ROPO}}_{3}^{2-}$ and ROPO_3_H^−^ compounds ν_as_(${\text{PO}}_{3}^{2-}$)	Also consistent with Lipid ν_s_(CO–O–C)
1033^*^	1025^*^	1042^*^	–	1031^*^	1032^*^	–	–	Phospholipid ν_as_(P–O–C)	Also consistent with saccharide primary alcohol ν(C–O)
–	–	–	1017^*^	1011^*^	1017^*^	1006^*^	1006^*^	Saccharide primary alcohol ν(C–O)	Also consistent with ν_s_(${\text{PO}}_{3}^{2-}$) in ROPO_3_H^−^ compounds
– 963^*^	– 964^*^	979 976^*^	– 966^*^	– 966^*^	– 965^*^	– 967^*^	– 966^*^	${\text{ROPO}}_{3}^{2-}$ compounds ν_s_(${\text{PO}}_{3}^{2-}$) Polysaccharide β-pyranose ring vibration	May be overlapped with a weak lipid ν_as_(C–N)
920 918^*^	919 919^*^	– 917^*^	923 919^*^	919 919^*^	920 919^*^	– 919^*^	916 918^*^	Polysaccharide pyranose O_as_(ring)	
– 886^*^	– 887^*^	– –	– 891^*^	– 889^*^	– 890^*^	888 887^*^	889 888^*^	Polysaccharide β-pyranose μ(C–H) Amino acid ν_s_(N–C–C)	
823 828^*^	– 827^*^	– –	826 829^*^	823 828^*^	824 829^*^	810 816^*^	811 814^*^	Phospholipid ν_as_(P–O)	Probably overlapped with ω(O–H/N–H) bands
765 769^*^	764 768^*^	– 773^*^	766 770^*^	766 769^*^	766 769^*^	– 770^*^	– 768^*^	Polysaccharide pyranose O_s_(ring)	Probably overlapped with ω(O–H/N–H) bands
723 730^*^	– 729^*^	–	724 730^*^	725 729^*^	725 729^*^	728^*^	728^*^	Lipid >CH_2_ ρ(C–H)	May be overlapped with a weak protein amide V band due to δ(N–H) and ω(O–H/N–H) bands
701 701^*^	701 701^*^	697 701^*^	701 701^*^	701 701^*^	700 701^*^	700 701^*^	700 701^*^	Polyglycine ρ(CH_2_) and μ(N–H) Polysaccharide ν(ring)	Also consistent with out-of-plane μ(N–H) in hydrogen-bonded secondary amides and Probably overlapped with ω(O–H/N–H) bands
659 659^*^	658 659^*^	658 656^*^	659 659^*^	659 660^*^	660 660^*^	659 659^*^	659 659^*^	Fatty acid aliphatic carboxylic acid μ(O–CO)	Probably overlapped with ω(O–H/N–H) bands and the δ(CO_2_) spurious band (~670 cm^−1^)
618 620^*^	617 620^*^	621 619^*^	613 620^*^	621 622^*^	618 621^*^	607 603^*^/623^*^	615 618^*^	Fatty acid aliphatic carboxylic acid μ(O–CO)	Probably overlapped with ω(O–H/N–H) bands
531^*^	531^*^	–	528^*^	524^*^	525^*^	529^*^	530^*^	Amino / Fatty acid aliphatic carboxylic acid μ(C–CO)	Probably overlapped with ω(O–H/N–H) bands
439 431^*^	– 431^*^	– 428^*^	435 430^*^	434 429^*^	435 430^*^	– 431^*^	– 429^*^	–	Possibly secondary amine δ(C–N–C), α-aliphatic carboxylic acid μ(C–CO), aliphatic ether μ(C–O–C), or a band from Fatty acid alkyl esters

^*a*^ Referring to the peak wavenumbers indicated in cm^−1^. The given band positions were rounded to the nearest unit from the FTIR measurements performed with a wavenumber step of 1 cm^−1^; hence, the accuracy of the band positions and shifts is 1 and 2 cm^−1^, respectively. ^*b*^ ν, stretching; μ, deformation; δ, bending; sc, scissoring; ρ, rocking; o, breathing; ω, wagging; τ, twisting; s, symmetric; as, asymmetric. ^*^ Estimated band positions for shoulder, hidden (due to overlapping), and observed bands (for comparison) using second derivatives of FTIR spectra.

**Figure 3:**
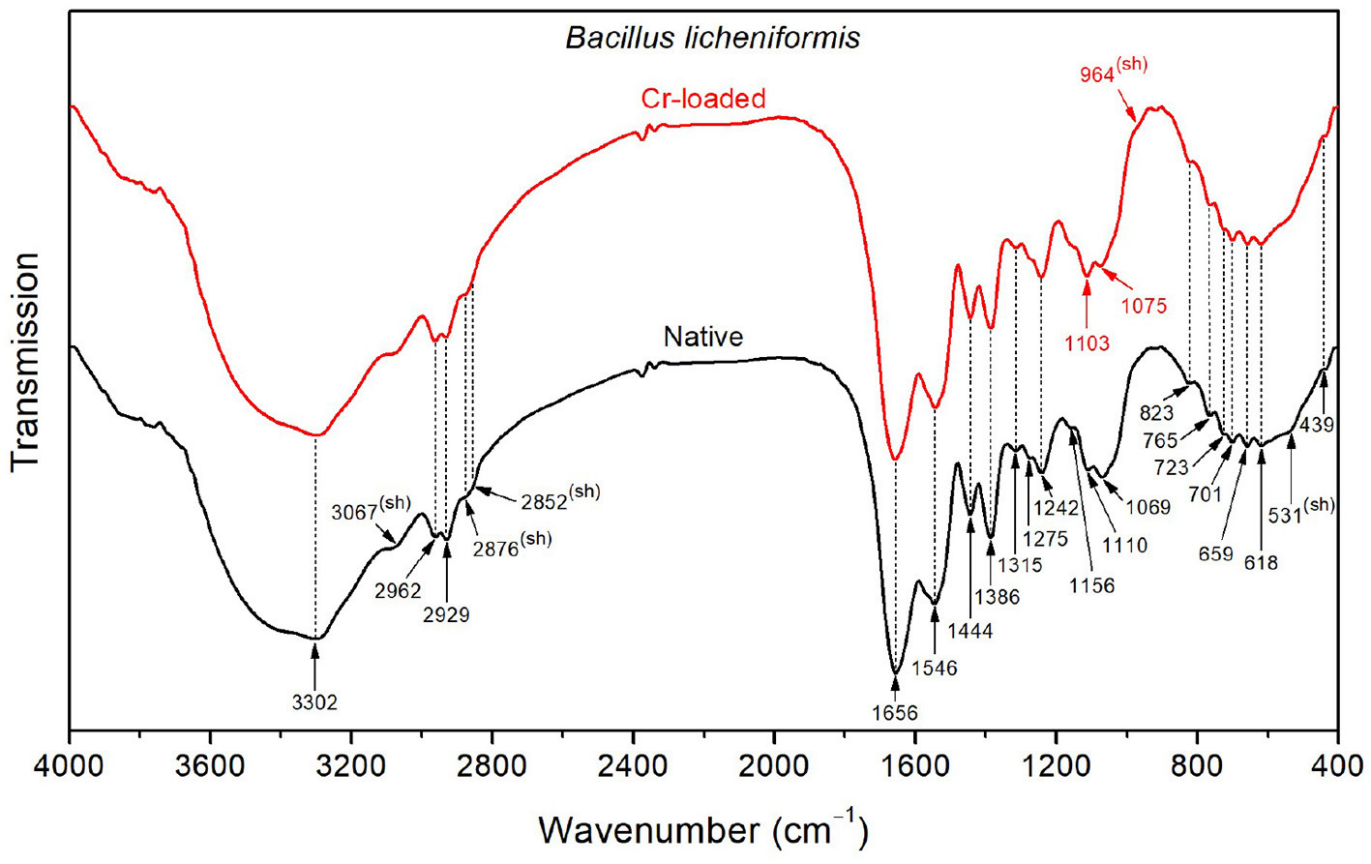
FTIR spectra of the *Bacillus licheniformis* bacterial strains before Cr loading (native) and after treatment with 50 mg/L Cr^6+^ (Cr-loaded).

As depicted in Figure 3, the FTIR spectrum of the native *Bacillus licheniformis* strains shows a prominent and asymmetric absorption band peaking at 3302 cm^−1^ due to protein and peptide N–H stretching (amide A band), which overlaps with a broad band related to O–H stretching from hydroxyl groups in carbohydrates (~3550–3150 cm^−1^), along with the ${\text{NH}}_{3}^{+}$ asymmetric stretching band from amino acids (~3200–3000 cm^−1^). Two strong secondary polyamide bands can be seen at ~1656 and 1546 cm^−1^. The vibration at 1656 cm^−1^ is ascribed to the amide I band in α-helical structures (proteins and polypeptides), which is dominated by C=O stretching (Venyaminov and Kalnin, 1990b; Socrates, 2001). In this regard, it is noteworthy to mention that C=C stretching in lipid esters (expected at about 1650 cm^−1^), β-type secondary structures of proteins (indiscernible features), along with ${\text{CO}}_{2}^{-}$ and ${\text{NH}}_{3}^{+}$ vibrations in amino acids (around 1686 and 1636 cm^−1^), may have contributed to the above-assigned amide I band (Venyaminov and Kalnin, 1990a,b; Naumann, 2000; Socrates, 2001). The band at 1546 cm^−1^ corresponds to the amide II band originating from in-plane N–H bending and C–N stretching and gives rise to an overtone feature, as shown by the shoulder around 3090 cm^−1^ (~3067 cm^−1^ as determined using the second derivative).

Moreover, the CH_3_ and CH_2_ functional groups (fatty acid chains in lipids) exhibit characteristic C–H asymmetric stretching vibrations at 2962 and 2929 cm^−1^, respectively. The corresponding C–H asymmetric stretching vibrations were resolved into components at 2876 and 2852 cm^−1^, respectively, yielding a single shoulder-like feature at about 2860 cm^−1^. These aliphatic groups are also seen at 1444, 1386, and 723 cm^−1^, corresponding to CH_2_ deformation, CH_3_ symmetric bending, and CH_2_ rocking characteristic bands, respectively (Socrates, 2001). The FTIR spectra also display weak features corresponding to the amide III band in α-helical protein and peptide structures (caused by coupled C–N stretching and N–H bending) and the amide V band (originating from N–H bending), as shown by the shoulders at ~1275 and 726 cm^−1^, respectively.

Characteristic bands of phosphodiester, free phosphate, and polysaccharide functional groups are found in the 1250–900 cm^−1^ region. Indeed, the bands at 1242 and 1110 cm^−1^ are attributed to the phosphodiester stretching modes, while the band at 1069 cm^−1^ refers to free phosphate ionic species. However, the latter is also consistent with the stretching of C–O and C–C bonds in polysaccharide rings and C–O–C bonds in lipids (Wilson et al., 2000; Socrates, 2001). Similarly, the band at 1156 cm^−1^ is consistent with C–O–C stretching in lipids, C–O stretching in monosaccharide compounds (such as glucose), and C–O–C stretching of the glycosidic link in polysaccharides (Naumann, 2000; Socrates, 2001). The bands at 765 and 920 cm^−1^ also refer to symmetric and asymmetric breathing of the pyranose ring in polysaccharide compounds (Copíková et al., 2001; Socrates, 2001). In this regard, the vibration at 886 cm^−1^ is characteristic of the anomer C–H deformation in carbohydrates and provides evidence of the major β-form of the polysaccharide pyranose rings (Socrates, 2001; Hong et al., 2021).

In the wavenumber range below 900 cm^−1^, weak-to-medium intensity features are overlapped with a broad medium-to-strong band centered at roughly 660 cm^−1^. The latter is typical of hydroxyl group O–H wagging, but it can also include contributions from other hydrogen-bonded groups, such as water O–H or amide N–H wagging motions. The presence of α-amino-acids (such as glutamic acid and alanine) in the *Bacillus licheniformis* spore is supported by features related to O–CO (bands at 659 and 618 cm^−1^) and C–CO (shoulder at 531 cm^−1^) deformation vibrations in α-aliphatic carboxylic acids in this spectral range (Hughes, 1968). The band at 439 cm^−1^ cannot be restrainedly assigned by cross-referencing with other bands and is not used in the discussion hereafter.

The FTIR spectrum of native *Bacillus licheniformis* also includes hidden vibrations, such as the band at 1749 cm^−1^ originating from C=O stretching of carboxylic groups (esters and fatty acids), the band at 1515 cm^−1^ related to C–C stretching in the tyrosine aromatic ring (side-chain vibration in amino acids), or the band at 1463 cm^−1^ due to methyl and methylene vibrations (Schmitt and Flemming, 1998; Socrates, 2001; Tremmel et al., 2005; El-Naggar et al., 2020).

The broad weak band at around 2120 cm^−1^, which overlaps with the water scissoring and rocking vibrations, is related to amino acid ${\text{NH}}_{3}^{+}$ symmetric stretching (Socrates, 2001; Lasagabaster et al., 2006). Close to this band, the absorption features at 2375 and 2340 cm^−1^ correspond to asymmetric stretching of adsorbed and gaseous CO_2_, respectively (Busca and Lorenzelli, 1982; Seiferth et al., 1999) and are most likely brought on by residual contamination.

The Cr(VI) treatment brings about the rise of the shoulder band (~964 cm^−1^) and the shift (from 1069 to 1075 cm^−1^) of the ${\text{PO}}_{3}^{2-}$ symmetric and asymmetric stretching bands, respectively. Furthermore, the ${\text{PO}}_{2}^{-}$ symmetric stretching band exhibits an increase in intensity along with a slight shift (from 1110 to 1113 cm^−1^). These changes suggest that the phosphate-containing compounds are involved in the interaction with metal ions. This interaction yields the emergence of negative phosphoryl groups as a result of a deprotonation process. This is further supported by the partial vanishing and the shift (from 1033 to 1025 cm^−1^) of the P–O–C stretching shoulder band. In addition, the increase of the ${\text{PO}}_{2}^{-}$ band (~1110 cm^−1^) is related to the observed weakening of the CH_3_ stretching, as shown by the decrease in intensity of the band at 2929 cm^−1^ (with respect to the CH_2_ band). This is also substantiated by the relative decrease of the CH_3_ bending band (1386 cm^−1^) with respect to the CH_3_ deformation band (~1444 cm^−1^) (Barkleit et al., 2011).

**Figure 4:**
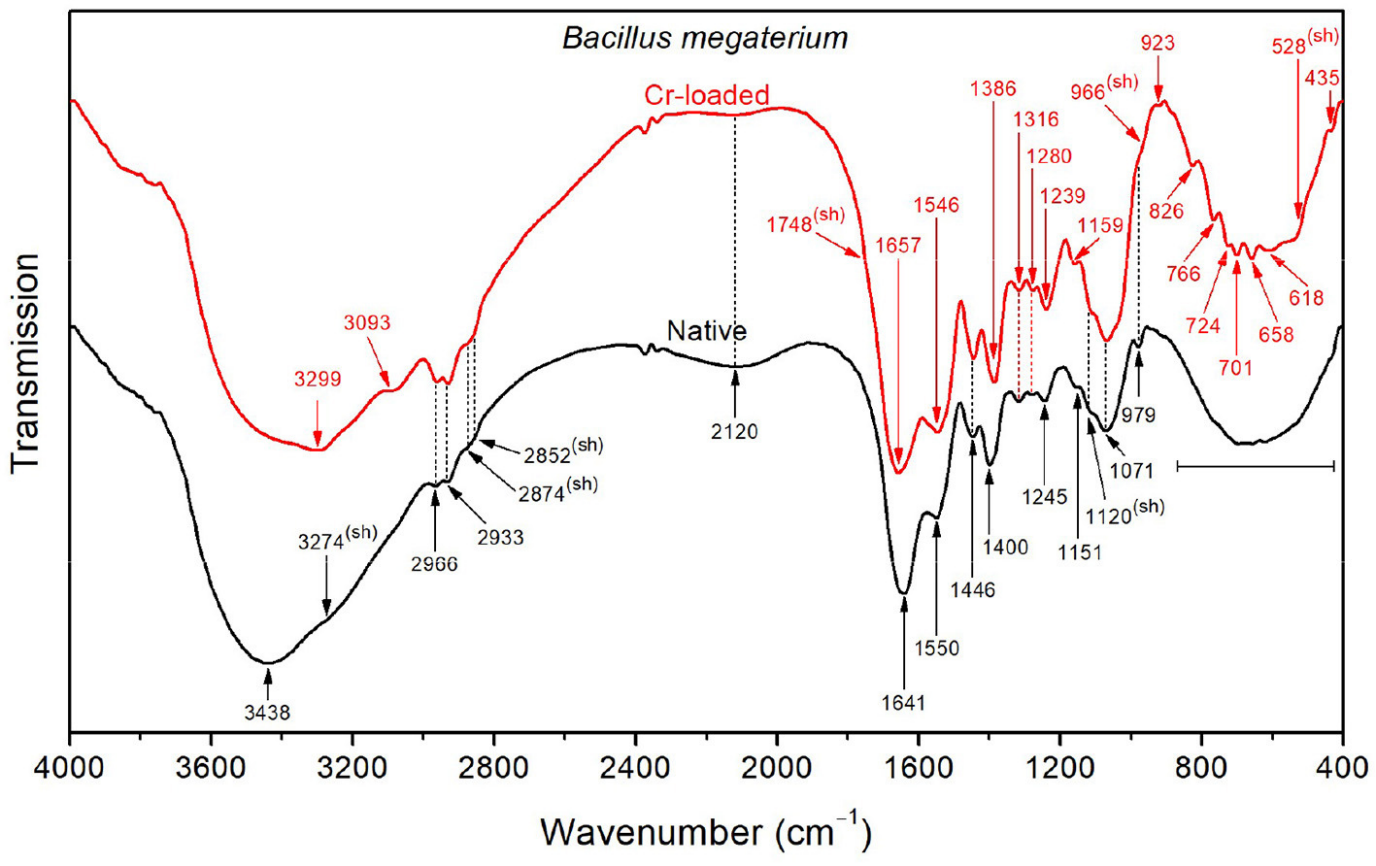
FTIR spectra of the native and Cr-treated (with 50 mg/L Cr^6+^) *Bacillus megaterium* bacterial strains.

In contrast to *Bacillus licheniformis*, the high-frequency region in the native *Bacillus megaterium* spectrum (Figure 4) is dominated by the OH stretching band (3438 cm^−1^) superimposed with the NH stretching band (shoulder at 3274 cm^−1^). This is also visible at low frequencies, where the broad OH wagging (400–900 cm^−1^) fully dominates the cluster of characteristic polysaccharide and amino acid absorption bands in this range. These intense OH vibrations probably stem from the monosaccharide content (mainly composed of D-glucose, D-xylose, D-galactose, and L-arabinose) in the *Bacillus megaterium* capsule (Cassity and Kolodziej, 1984). Additionally, the amide I and II bands appear at shifted positions (1641 and 1550 cm^−1^, respectively) as a result of strong stretching of ${\text{NH}}_{3}^{+}$ and COO^−^ in amino acids (Venyaminov and Kalnin, 1990a). This is in agreement with the medium and broad band observed at 2120 cm^−1^ originating from ${\text{NH}}_{3}^{+}$ asymmetric stretching and the COO^−^ band at 1400 cm^−1^. Therefore, a broad medium band arising from ${\text{NH}}_{3}^{+}$ symmetric stretching in the 2760–2530 cm^−1^ region could explain the weak intensity of CH_2_ and CH_3_ bands and shoulders observed at 2966, 2933, 2874, and 2852 cm^−1^.

Upon exposure of the *Bacillus megaterium* strains to Cr(VI), the intense OH stretching band undergoes a significant decrease in intensity, revealing the underlying NH stretching band at 3299 cm^−1^, which indicates that conformational changes occurred. Furthermore, the amide I and II bands appear at 1657 and 1546 cm^−1^, indicating the decrease of the amino acid ${\text{NH}}_{3}^{+}$ and ${\text{CO}}_{2}^{-}$ bands, as mentioned above. Accordingly, the CH_3_ bending band appears at 1387 cm^−1^ as a result of the weakened ${\text{CO}}_{2}^{-}$ symmetric stretching band. The emergence of a noticeable shoulder at 1748 cm^−1^ relative to C=O stretching in COOH functional groups also witnesses that the amino acids are involved in the interaction with chromium. The bands in the region below 900 cm^−1^ appear after chromium treatment as a result of the weakening of presumed OH wagging. However, the overall absorption in the 1800–900 cm^−1^ region increases after treatment with chromium, which could be tentatively assigned to the emergence of overtone features related to NH_2_ wagging in the 900–400 cm^−1^ region.

**Figure 5:**
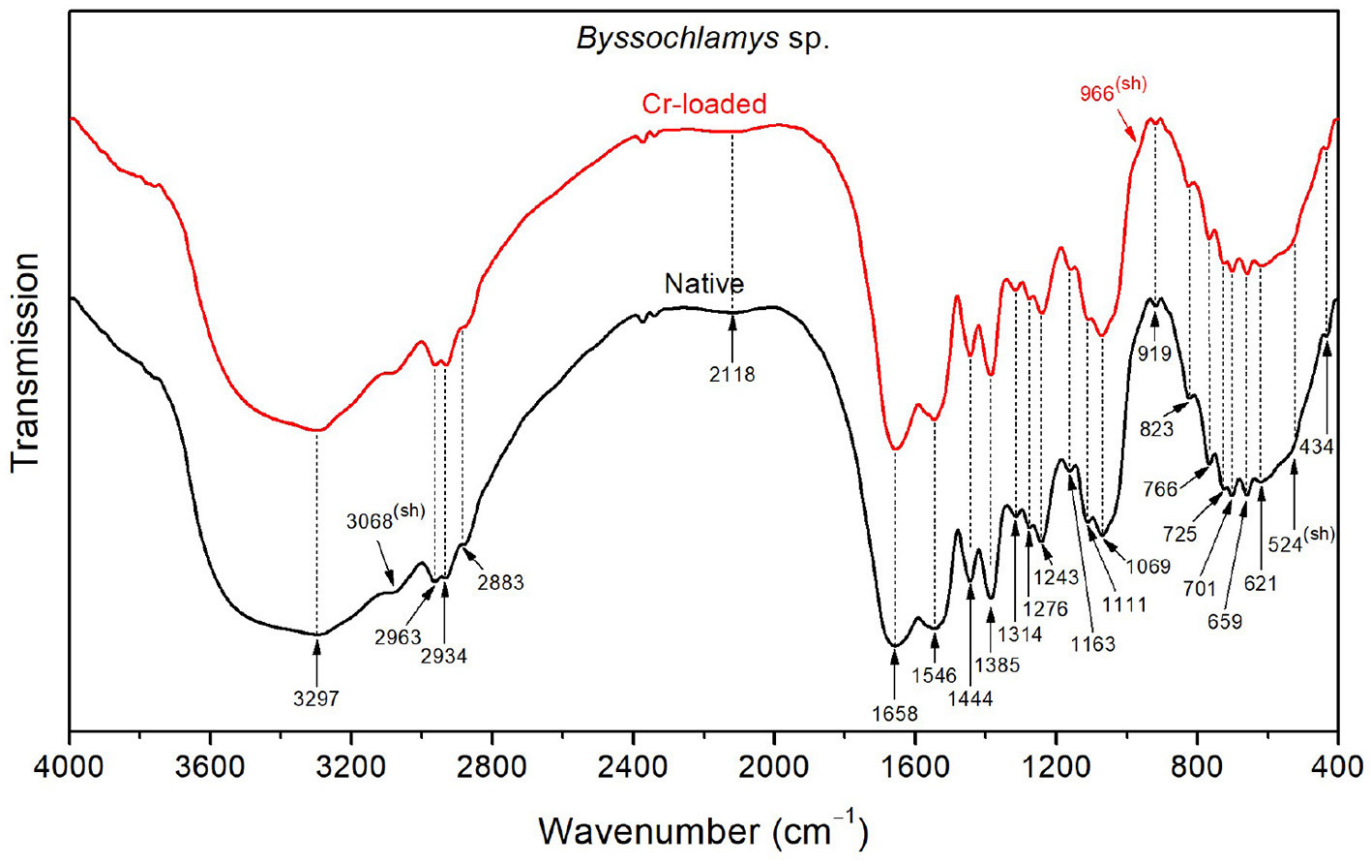
FTIR spectra of the *Byssochlamys* sp. strains before Cr loading (native) and after treatment with 50 mg/L Cr^6+^ (Cr-loaded).

As shown in Figure 5, the FTIR spectrum of the native *Byssochlamys* sp. strains exhibits typical vibrational bands as seen previously, including the overlapped OH and NH stretching bands peaking at 3297 cm^−1^, CH_2_ and CH_3_ asymmetric stretching bands at 2963 and 2934 cm^−1^, and the characteristic vibrations of carbohydrates and lipids in the 900–400 cm^−1^ spectral region (Table 2). The main specificities lie in the broadened amide bands at 1658 and 1546 cm^−1^, most likely due to motions of the carboxylate and ammonium functional groups, as previously explained.

After treatment with chromium, the high-frequency OH stretching band (~3400 cm^−1^) and the low-frequency OH wagging band (900–400 cm^−1^) slightly decreased in intensity. Additionally, the side of the amide I band toward low frequencies (i.e., lower than 1658 cm^−1^) and that of the amide II band toward high frequencies (i.e., <1546 cm^−1^) become narrower, which indicates reduced contributions from the ${\text{NH}}_{3}^{+}$ asymmetric deformation (found at 1626 and 1572 cm^−1^ for the treated strains).

**Figure 6:**
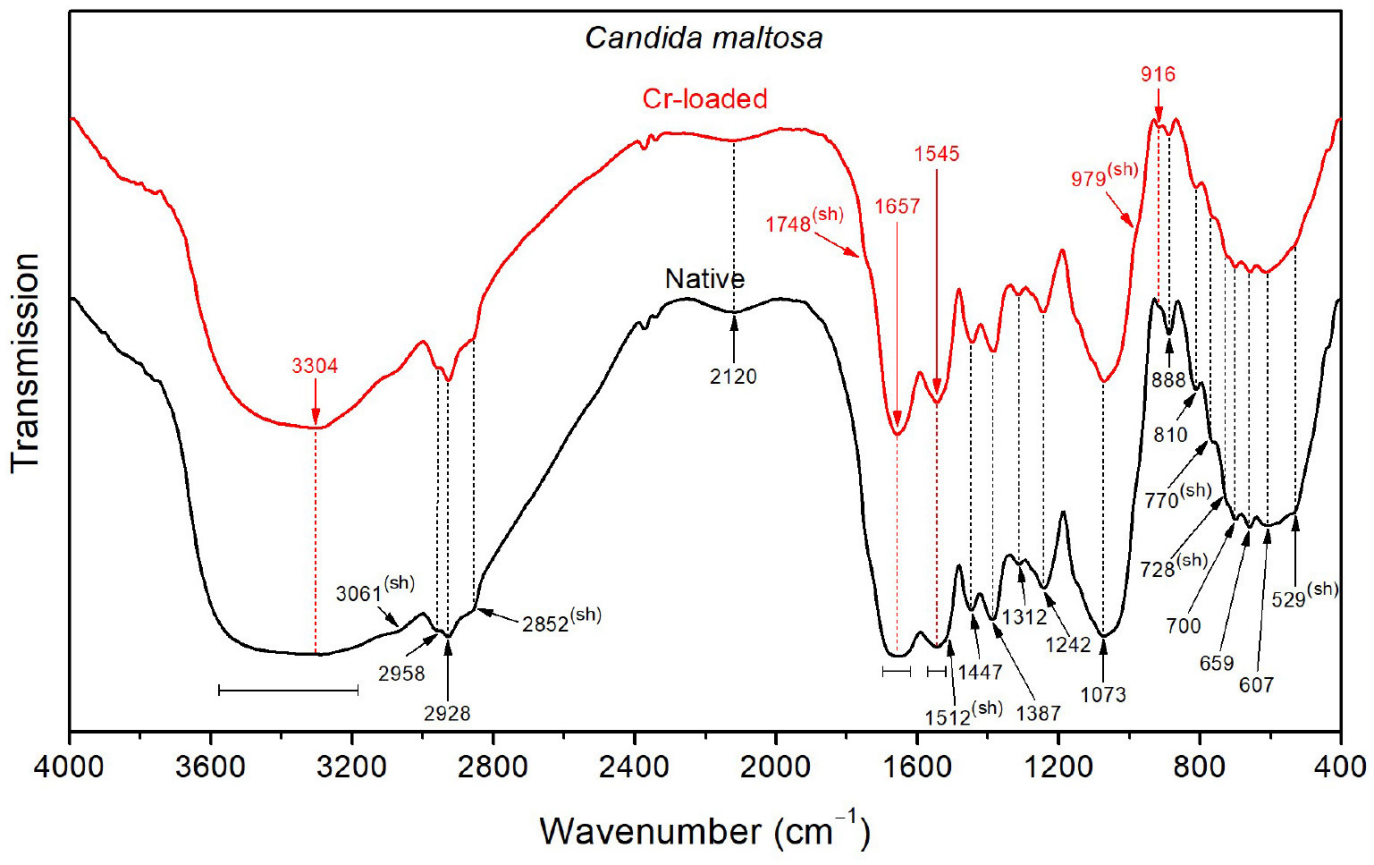
FTIR spectra of the *Candida maltosa* strains before Cr loading (native) and after treatment with 50 mg/L Cr^6+^ (Cr-loaded).

With respect to *Bacillus licheniformis*, the spectrum of the native *Candida maltosa* strains (Figure 6) exhibits an intense broad O–H stretching band in the region 3650–3550 cm^−1^, along with a stronger O–H wagging in the region 900–400 cm^−1^. This is likely to arise from the hydroxyl-rich glycosylphosphatidylinositol in the outer layer (mannose-containing compounds) and the polysaccharide-rich inner layer (β-glucan and chitin) in the cell wall of *Candida* strains (Chaffin et al., 1998; Kapteyn et al., 2000; Gow et al., 2011; Zvonarev et al., 2021). Accordingly, the methylene groups (abundant in the phospholipid tail of glycosylphosphatidylinositol) show a stronger absorption with respect to the methyl groups, as shown by the asymmetric stretching bands (2928 and 2958 cm^−1^, respectively) and the sharpened shoulder band at 2852 cm^−1^ (i.e., at the position of symmetric stretching of CH_2_). This is also consistent with the strong absorption in the spectral ranges 1200–950 cm^−1^ due to the C–O stretching in polysaccharide C–OH and C–O–C branches (mannans) and 1400–1300 cm^−1^ due to carbohydrate OH and CH deformation vibrations (see Table 2). Besides, the amide I band (~1655 cm^−1^) is relatively broad, probably due to overlapping with the ${\text{CO}}_{2}^{-}$ stretching features (~1697 and 1621 cm^−1^).

The FTIR spectrum shows a decrease in the intensity of the OH stretching band after loading with Cr(VI), suggesting that hydroxyl groups may be involved in the interaction with metallic ions. In addition, the shoulder relative to C=O stretching of –COOH functional groups (~1748 cm^−1^) becomes more noticeable after chromium treatment. The narrower amide I band (~1657 cm^−1^) for the treated strain probably originates from the partial vanishing of the ${\text{CO}}_{2}^{-}$ stretching modes, which were also shifted (from 1697 to 1691 cm^−1^ and from 1621 to 1626 cm^−1^). The same effect can be seen for the amide II band (~1545 cm^−1^), probably due to the decrease of ${\text{NH}}_{3}^{+}$ deformation vibrations, as shown by the softened shoulder band at 1512 cm^−1^. This is further supported by the decrease in intensity of the ${\text{NH}}_{3}^{+}$ stretching band at 2120 cm^−1^ upon chromium treatment.

### 3.4 Zeta potential analysis

The zeta potential was measured for the four microbial strains in the absence and presence of different Cr^6+^ concentrations ranging from 25 to 800 mg/L (Table 3). Before contact with Cr^6+^, the microbial strains show different zeta potentials of about −10.63 ± 0.38, −15.9 ± 1.39, −8.98 ± 0.40, and −10.15 ± 2.68 mV for *Bacillus licheniformis, Bacillus megaterium, Byssochlamys* sp., and *Candida maltosa*, respectively. These negative potential differences with respect to the medium are related to the amounts of negative charges present at the vicinity of the microorganisms before being brought into contact with Cr^6+^.

**Table 3:** Zeta potential of *Bacillus licheniformis, Bacillus megaterium, Byssochlamys* sp., and *Candida maltosa* strains in the absence and presence of different concentrations of Cr^6+^.

**Cr^6+^ concentration (mg/L)**	**Zeta potential (mV)**				
	***Bacillus licheniformis***	***Bacillus megaterium***	***Byssochlamys*** **sp**.	***Candida maltosa***	**Control**
0	−10.63 ± 0.38^c^	– 15.9 ± 1.39^b^	– 8.98 ± 0.40^c^	– 10.15 ± 2.68^c^	– 12.15 ± 2.93^cd^
25	– 26.53 ± 0.29^b^	– 29.37 ± 0.55^a^	– 18.33 ± 4.40^ab^	– 13.63 ± 0.15^bc^	– 17.07 ± 2.67^bc^
50	– 24.37 ± 0.60^b^	– 28.47 ± 0.38^a^	– 20.7 ± 0.98^a^	– 17.73 ± 1.08^ab^	– 18.53 ± 0.06^b^
100	– 39.53 ± 1^a^	– 30.4 ± 1.51^a^	– 20.53 ± 1.04^a^	– 21.93 ± 2.08^a^	– 35.3 ± 0.62^a^
250	– 26.23 ± 1.10^b^	– 22.33 ± 2.04^ab^	– 13.70 ± 0.00^abc^	– 10.78 ± 2.44^c^	– 9.81 ± 2.39^d^
500	– 22.7 ± 1.97^b^	– 15.3 ± 2.18^b^	– 16.07 ± 6.14^abc^	– 11.29 ± 3.14^c^	– 3.87 ± 1.05^e^
800	– 25.37 ± 3^b^	– 16.97 ± 8.36^b^	– 12.23 ± 0.75^bc^	– 12.13 ± 1.66^bc^	– 3.75 ± 0.31^e^

^a, b, c, d, e^indicate statistically significant differences (*p* < 0.05) of the turkey test among different zeta potential values.

After exposure to chromium, the zeta potential of *Bacillus licheniformis* strains undergoes a significant change from −10.63 ± 0.38 (0 mg/L Cr^6+^) to −39.53 ± 1 mV (100 mg/L Cr^6+^), indicating that the cells in contact with Cr^6+^ have experienced an increase in negative charge. As the Cr^6+^ concentration increases in *Bacillus licheniformis*, the zeta potentials are statistically identical up to 100 mg/L. The zeta potential reaches the highest value (−39.53 ± 1 mV) at 100 mg/L before decreasing beyond that.

For *Bacillus megaterium*, the zeta potential range from −15.3 ± 2.18 to −30.4 ± 1.51 mV for the investigated Cr^6+^ concentrations. Significant differences are also observed between the control and the strains treated with different concentrations of Cr^6+^ (from 25 to 250 mg/L).

The Cr^6+^-treated *Byssochlamys* sp. show zeta potentials that are statistically different from those of the control. The zeta potentials are between −8.98 ± 0.40 and −20.7 ± 0.98 mV and correspond to the values for the Cr^6+^ concentrations of 0 and 50 mg/L. Statistically, the highest zeta potential values are −20.7 ± 0.98 and −20.53 ± 1.04 mV (50 and 100 mg/L of Cr^6+^). In contrast, the lowest value is recorded for the control (−8.98 ± 0.40 mV).

Finally, zeta potentials that are statistically different from the control were recorded for the Cr^6+^-treated *Candida maltosa* strains. This concerns the zeta potentials of treatments with 25, 50, 100, and 800 mg/L Cr^6+^. The zeta potential values range from −10.15 ± 2.68 to −21.93 ± 2.08 mV (control and 100 mg/L Cr^6+^).

## 4 Discussion

Biosorption (or bioadsorption) is a passive, rapid, and reversible physico-chemical phenomenon between a metal and a biological material (biosorbent) (Fernandez et al., 2018). It independent from cell activity carried out by active or inactive microorganisms (Fernandez et al., 2018). This process was assessed using SEM-EDX, FTIR analysis, and zeta potential measurements. Adsorption relies on hexavalent (or trivalent chromium produced by hexavalent chromium reduction) binding through a number of molecules. Biosorption is also the passive immobilization of metals by biomass. Sorption mechanisms at the cell surface take place independently of the cell's metabolism. These mechanisms are based on physico-chemical interactions between the metal and functional groups in the cell wall (Kaduková and Virčíková, 2005).

### 4.1 SEM analysis

The morphology of the microbial strains analyzed by SEM showed different characteristics. The *Bacillus licheniformis* strains have a smooth surface in the control and after treatment with 5 mg/L Cr^6+^. Similar results obtained by Hossan et al. (2020) showed that *Klebsiella* sp. have a smooth surface in the control cells. This smooth structure in untreated cells has also been observed in previous studies (Karthik et al., 2017; Bharagava and Mishra, 2018; Zhu et al., 2019; Kalola and Desai, 2020; Tan et al., 2020; Abo-Alkasem et al., 2022). Jobby et al. (2019), Selvakumar et al. (2021), and Su et al. (2021) demonstrated that the cells of *Sinorhizobium* sp. SAR1, *Bacillus vietnamensis, Bacillus lentus, Alcaligenes faecalis, Staphylococcus cohnii, Staphylococcus saprophyticus*, and *Rhodobacter sphaeroides* SC01 also have smooth surfaces in the absence of Cr^6+^. Additionally, intact and regular cells of *Bacillus licheniformis* were observed in the absence of metal. This case is similar to that found by Sevak et al. (2023) in *Acinetobacter* sp. biomass. Hossan et al. (2020) observed depression in *Klebsiella* sp. treated with 100 mg/L Cr^6+^. After exposure to 5 mg/L Cr^6+^, the biomass of *Bacillus licheniformis* retains its smooth structure and the metal does not damage the cells, likely indicating the effective resistance of the cell at this concentration level. The wrinkles, rough clusters, irregular shapes, and possible production of extracellular substances in the biomass treated at higher concentrations (50 mg/L Cr^6+^) revealed that the cells develops a strategy to circumvent the toxic effect of Cr^6+^. Elahi et al. (2022) described wrinkles in *Bacillus cereus* strain b-525k after contact with 2 mM Cr^6+^. Similarly, Hossan et al. (2020) reported ruptured surfaces in the biomass of *Klebsiella* sp. after treatment with Cr^6+^.

The *Bacillus megaterium* cells were not individually distinguishable with and without Cr^6+^ in the medium. Hossan et al. (2020) noted a similar case in *Klebsiella* sp. control. In addition, the irregular appearance observed in *Bacillus megaterium* in media with or without Cr^6+^ is a common phenomenon in microorganisms. This could be due to the secretion of extracellular substances around the cells. These extracellular substances are probably reduced in cells grown at 50 mg/L Cr^6+^ because of the toxic effect of the metal and could be exopolysaccharides. These research findings are in accordance with earlier studies (Ozturk et al., 2009; Jobby et al., 2019).

The *Byssochlamys* sp. control biomass and that treated with 5 mg/L Cr^6+^ present identical aspects. The concentration of 5 mg/L Cr^6+^ shows no impact on the cells, which remain smooth and less bound. However, at 50 mg/L Cr^6+^, the cells group together to provide a better Cr^6+^ adsorption surface. In this regard, Majumder et al. (2017) found ruptured surfaces with Cr^6+^ saturation on the biomass of *Arthrinium malaysianum*.

The *Candida maltosa* control cells and those treated with 5 mg/L Cr^6+^ appear in clusters with smooth outlines. However, at 5 mg/L Cr^6+^, some cells are less visible and form irregular blocks. This phenomenon is thought to be due to the production of extracellular substances to resist the toxic effect of Cr^6+^. At 50 mg/L Cr^6+^, several cells are unable to retain their initial structures probably due to damaged cellular constituents of strains, as a result of the toxic effect of Cr^6+^ (Mat Arisah et al., 2021).

In the literature, some authors mentioned that wrinkles appeared on the biomass of *Aspergillus terricola* after treatment with Cr^6+^ (Mohamed et al., 2021). In the absence of Cr^6+^ in the medium, Dwivedi (2023) found that the hyphae of *Talaromyces pinophillus* are thin, rough, and loose. However, with Cr^6+^ stress, the hyphae are aggregated and dense. Similarly, Saranya et al. (2020) observed the same rough structures on *Trichoderma asperellum* after contact with Cr^6+^ and indicated the adsorption of Cr^6+^ and Cr^3+^.

Majumder et al. (2017) showed that the cell surfaces are saturated by the toxic agent, and Mat Arisah et al. (2021) explain that the rough surfaces induced by Cr in the treatments are due to the adsorption of Cr^6+^.

### 4.2 EDX analysis

EDX analysis showed the presence of peaks in all the biomasses stressed with Cr^6+^. However, the biomasses not treated with Cr showed no chromium peaks. The appearance of Cr peaks in the biomasses treated with 5 and 50 mg/L Cr^6+^ indicates the presence of Cr bound to the cell surfaces of the strains (Karthik et al., 2017). The drop in the weight percentage of Cr is thought to be due to Cr toxicity, which damages parts of the cell surfaces (Su et al., 2021). Indeed, Cr is a metal capable of causing cell lysis and therefore bacteria and fungi could biosorb it. In this respect, Majumder et al. (2017) found that chromium could bind to the dried biomass of the fungus *Arthrinium malaysianum* (Cr peak detection). Similarly, Karthik et al. (2017) studied the ability of *Cellulosimicrobium funkei* strain AR6 to adsorb Cr at concentrations ranging from 100 to 250 μg/mL. They attributed the presence of Cr peaks to Cr^3+^ (reduction of Cr^6+^) bound to cell surfaces. Kalola and Desai (2020) pointed out that Cr adsorption also occurs in *Halomonas* sp. DK4. Additionally, Cr-specific peaks were detected in *Bacillus vietnamensis, Bacillus lentus, Alcaligenes faecalis, Staphylococcus cohnii*, and *Staphylococcus saprophyticus* treated with 3400 mg/L Cr^6+^ (Selvakumar et al., 2021). Su et al. (2021) and Kalsoom A. et al. (2021) indicated that the presence of Cr peaks could be due to the complexation of Cr^3+^ ions with molecules located on the cell surface or the presence of reduced Cr^3+^ species precipitated on the outer surface of the strains. The peak observed by the scientists (Kalsoom A. et al., 2021) was in the biomass of *Staphylococcus simulans* treated with 1500 μg/mL Cr^6+^ (0.19 wt.% Cr).

The weight percentage of chromium detected in *Salipaludibacillus agaradhaerens* strain NRC-R stressed with 4 mM Cr^6+^ was 0.16 % (Abo-Alkasem et al., 2022). Moreover, Elahi et al. (2022) showed that adsorption is one of the detoxification mechanisms of *Bacillus cereus* b-525k.

Microcharacterization of *Cellulosimicrobium* sp. (SCRB10), *Bacillus* sp. CRB-B1, and *Klebsiella* sp. treated with 100, 150, and 100 mg/L Cr^6+^, respectively, revealed Cr adsorption with weight percentages equivalent to 0.71, 3.54, and 6.02, respectively (Bharagava and Mishra, 2018; Hossan et al., 2020; Tan et al., 2020). These findings are in contrast with the results of some scientists showing that some strains do not adsorb Cr. For instance, Jobby et al. (2019) and Sevak et al. (2023) demonstrated that *Sinorhizobium* sp. SAR1 and *Acinetobacter junii* strain b2w do not adsorb Cr after contact with metal.

### 4.3 FTIR analysis

FTIR can be used to detect the functional groups that allow the adsorption of Cr. The chromium adsorption mechanism was carried out in a culture medium enriched with Cr^6+^ in order to identify the functional groups that appear only in the presence of Cr^6+^. This gave us a clear understanding of Cr^6+^ bioremediation. The presence of other metals would have obscured this understanding.

Filamentous fungi are capable of adsorbing Cr^6+^ and this ability has been demonstrated by several researchers (Shroff and Vaidya, 2012; Majumder et al., 2017). The absorption peaks show molecules, such as NH, CH_3_, CH_2_, C=O, C=N, OH, PO^2−^, P–O, and O–CO in *Bacillus licheniformis* control and exposed to Cr^6+^. This suggests that these molecules are involved in the binding of Cr^6+^ or Cr^3+^ (Ayele et al., 2021; Su et al., 2021; Elahi et al., 2022). The peaks that remain at 3067, 1275, 1156, 1110, 1069, and 531 cm^−1^ and that corresponding to amide II, amide III, C–O–C, PO^2−^, C–O, C–C, ${\text{PO}}_{3}^{2-}$, C–CO, or those that appear at 1075 cm^−1^ (C–O and C–C) and 964 cm^−1^ (${\text{PO}}_{3}^{2-}$) highlight the strain resistance to metal (Su et al., 2021).

Functional groups, such as CH_3_, CH_2_, ${\text{CH}}_{3}^{+}$, hydroxide (OH), amide III, PO^2−^, C–O, C–C, and ${\text{PO}}_{3}^{2-}$ found on the cell surfaces of *Bacillus megaterium* biomasses grown in the absence and presence of Cr^6+^ are involved in the binding of Cr^6+^ and Cr^3+^ ions (Ayele et al., 2021; Su et al., 2021; Elahi et al., 2022). O–H (3438 cm^−1^), amide A (3274 cm^−1^), ${\text{NH}}_{3}^{+}$ (1641 cm^−1^), N–H and C–N (1550 cm^−1^), ${\text{CO}}_{2}^{-}$ (1400 cm^−1^), PO^2−^ (1245 cm^−1^), and C–O–C (1151 cm^−1^) were mainly identified on the control cells. These molecules play an important role in Cr resistance. In cells in contact with Cr^6+^, amide A (3299 cm^−1^), C=O (1748 cm^−1^; 1657 cm^−1^), ${\text{CO}}_{2}^{-}$ (1546 cm^−1^), CH_3_ (1386 cm^−1^), PO^2−^ (1239 cm^−1^), C–O–C (1159 cm^−1^), polysaccharide β-pyranose ring vibration (966 cm^−1^), asymmetric breathing of polysaccharide pyranose (ring) (923 cm^−1^), P–O (826 cm^−1^), symmetric breathing of polysaccharide pyranose (766 cm^−1^), C–H (724 cm^−1^), CH_2_ and N–H (701 cm^−1^), O–CO (658 and 618 cm^−1^), and C–CO (528 cm^−1^) were found. This indicates that the strain increase its resistance to chromium by producing other molecules to adsorb metal ions.

*Byssochlamys* sp. cells bind Cr^6+^ and Cr^3+^ with amide A, CH_3_, CH_2_, OH, amide III, PO^2−^, C–O–C, C–O, C–C, polysaccharide, P–O, N–H, N–H_2_, O–CO, and C–CO (Ayele et al., 2021; Su et al., 2021; Elahi et al., 2022). It was observed that a peak at 3068 cm^−1^ indicates the presence of the amide II. In addition, polysaccharide β-pyranose ring vibration (966 cm^−1^) detected in Cr^6+^-treated cells suggests that the polysaccharide produced adsorbs Cr in its various forms (Karthik et al., 2017; Ayele et al., 2021; Su et al., 2021; Elahi et al., 2022).

On the other hand, CH_3_, CH_2_, ${\text{NH}}_{3}^{+}$, amide I, amide II, OH, PO^2−^, C–O, C–C, polysaccharide, C–H, P–O, O–CO, and C–CO recorded on the *Candida maltosa* cell surface reveal their involvement in metal biding (Ayele et al., 2021; Su et al., 2021; Elahi et al., 2022). The *Candida maltosa* biomass control cells have cell surfaces composed of amide II (3061 cm^−1^) and ${\text{NH}}_{3}^{+}$ (1512 cm^−1^). In contrast, there was C=O (1748 cm^−1^) and ${\text{PO}}_{3}^{2-}$ (979 cm^−1^) in the biomass treated with Cr^6+^. The molecules produced during treatment are part of the strategy of resistance to the toxic metal.

The disappearance of certain functional groups observed at different frequencies is thought to be due to the toxic effect of Cr^6+^ (Karthik et al., 2017). The appearance of new functional groups in chromium-treated cells mean that the strain is setting up a resistance mechanism (Karthik et al., 2017; Princy et al., 2020). These molecules participate in the adsorption of Cr^6+^ and Cr^3+^ (Bharagava and Mishra, 2018). According to the results obtained, some functional groups remain after chromium treatment. This phenomenon is in line with certain studies carried out by scientists. Bharagava and Mishra (2018) noted that before and after treatment, there is always an asymmetric stretching of proteins, polysaccharides, lipids, and nucleic acids. Fungal cell surfaces interact with Cr and functional groups on these surfaces include carboxyl (COOH), phosphate (${\text{PO}}_{4}^{3-}$), amine (–NH_2_), thiol (–SH), and hydroxyl (–OH) groups (García-Hernández et al., 2017). The distinctive peaks observed on the cell surfaces of cells exposed to Cr are common in many scientific papers. This reflects the ability of microbial strains to sequester chromium on their cell surfaces (Jobby et al., 2019; Kalsoom A. et al., 2021; Mat Arisah et al., 2021; Sharma et al., 2022).

Karthik et al. (2017) indicated that several functional groups such as, O–H, C–H, and C–O, are involved in metal-microbial interaction. Bharagava and Mishra (2018) pointed out that *Cellulosimicrobium* sp. cells sequester Cr on their surface using molecules with specific groups, such as alkene (C=C), carbonyl (C=O), nitro (–NO_2_), carboxyl (–COOH), amines (–NH_2_), and sulphonic (–SO_3_).

It was revealed that amine (N–H), carboxyl (C=O), ether (C–O), alkyl halides (C–Cl, C–Br, and C–I), nitrile (C=N), and alkane (C–H) contribute to the retention of Cr on the cell surface of *Morganella morganii* (1Ab1) (Princy et al., 2020). Moreover, functional groups, such as O–H, N–H, C–HO, and C–O, were revealed on the *Exiguobacterium mexicanum* cell wall (Das et al., 2021).

Maurya et al. (2022) found –OH, –CH_2_, –COOH, C=O, –CH_3_, –COO^−^, –SO_3_H, S=O, C–OH, and C–O groups in *B. vallismortis, B. haynesii*, and *A. aquatilis* exposed to Cr^6+^. In addition, *Acinetobacter junii* strain b2w was shown to make use of –OH, C–H, C=O, C–N, C–O, and C–N functional groups to bond Cr (Sevak et al., 2023).

Once Cr^6+^ is bound to cell surfaces, it is reduced to Cr^3+^, which is less harmful. The microbial biomass can be recovered after centrifugation. The pellet was further washed with 1 mL of 10 mM EDTA solution for desorption of Cr from the cell surfaces. This method of decontamination is environmentally friendly. In addition, the biomass recovered can be used for another experiment in wastewater treatment.

### 4.4 Zeta potential analysis

Zeta potential analysis shows the different trends of charge present in tested microorganisms in the absence and presence of different hexavalent chromium concentrations. The presence of negative charges on cell surfaces is thought to be due to functional groups and that biosorption of Cr^6+^ modifies the zeta potential profiles. The attachment of chromium anion to cell surfaces leads to an accumulation of negative charges (Larson et al., 2023). The negative values of the zeta potentials confirm the accumulation of negative charges on the cell surfaces (Al-Jubory et al., 2020; El Malti et al., 2021). This observation suggests that electrostatic interactions play a role in metal adsorption. Modifications to the zeta potentials were observed by Huang et al. (2013), who indicated that the adsorption of cadmium (Cd) is due to electrostatic interactions. The authors found that living and dead *Bacillus cereus* RC−1 cells exhibit different zeta potentials in the presence of Cd(II). They noted that the negative charges on the surface of the dead cells are higher than those on the living cells. In this study, the negative charges varied from one strain to another. In *Bacillus licheniformis*, the highest negative charge of about −39.53 ± 1 mV was observed at 100 mg/L Cr^6+^. In *Bacillus megaterium* and *Candida maltosa*, the highest negative charges recorded at 100 mg/L Cr^6+^ were −30.4 ± 1.51 and −21.93 ± 2.08 mV, respectively. In *Byssochlamys* sp., the highest negative charges were found at 50 and 100 mg/L Cr^6+^ of about −20.7 ± 0.98 and −20.53 ± 1.04 mV, respectively. The difference in absolute load values between the control and the Cr^6+^ concentrations (25 to 800 mg/L) were 15.90, 13.74, 28.90, 15.60, 12.07, and 14.74 for *Bacillus licheniformis*. The difference in absolute load values between the control and the 25 to 800 mg/L Cr^6+^ concentrations were 13.47, 12.57, 14.50, 6.43, −0.60, and 1.07 in *Bacillus megaterium*. Charge differences of 9.35, 11.72, 11.55, 4.72, 7.09, and 3.25 were found at concentration values ranging from 25 to 800 mg/L Cr^6+^ in *Byssochlamys* sp. The differences in absolute load values for Cr^6+^ concentrations ranging from 25 to 800 mg/L were 3.48, 7.58, 11.78, 0.63, 1.14, and 1.98 for *Candida maltosa*. These results show that the Cr adsorption limit for all strains is 100 mg/L.

In a previous study, Srinath et al. (2002) investigated the ability of *Bacillus megaterium* to adsorb Cr. They demonstrated that living and dead cells of this strain adsorb 15.7 and 30.7 mg Cr/g dry weight, respectively. In contrast, 23.8 and 39.9 mg Cr/g dry weight were observed as Cr adsorption values in living and dead *Bacillus coagulans* cells. Other studies have shown that bacteria, yeasts, and filamentous fungi are capable of adsorbing Cr (Quintelas et al., 2008; Ahluwalia, 2014; Dadrasnia et al., 2015; Iram et al., 2015; Chang et al., 2016; Vendruscolo et al., 2017). In addition, Kaduková and Virčíková (2005) showed that biosorption is possible with both living and dead microorganisms with, however, a promoted activity for dead cells. *Pseudomonas* strains are known to adsorb Cr^6+^. It has been shown that *P. aeruginosa* 99, *P. stutzeri* T3, and *P. aeruginosa* 78 adsorb 22%, 14%, and 12% of 10 mg/L Cr onto the cell surfaces, respectively (Ozturk et al., 2012). The ability of a fungal consortium composed of *Cladosporeum perangustum* and *Penicillium commune* (isolated from tannery wastewater-contaminated soil), *Paecilomyces lilacinus*, and *Fusarium equiseti* (isolated from tannery sludge) to detoxify Cr^6+^-laden wastewater was revealed by Sharma and Malaviya (2016). The fungi were able to remove 100% of the Cr^6+^, with adsorption as the main mechanism. Singh et al. (2016) proved that *Aspergillus flavus* adsorb a maximum Cr^6+^ concentration equivalent to 16.1 mg/g. Bibi et al. (2018) mentioned that endophytic fungi could adsorb Cr^6+^ and act as fertilizers.

On the other hand, *Pseudomonas aeruginosa* RW9 was found to bind 0.46 mg/L Cr^6+^ to the cell surface (Mat Arisah et al., 2021). Other research works corroborate the results obtained in this study, i.e., the potential of fungi and bacteria in sequestering Cr on cell surfaces. Other microorganisms have been demonstrated to serve as effective agents in eliminating toxic metals. Additionally, Anupong et al. (2022) highlighted the ability of *Aspergillus flavus* DDN to eliminate the Cr contained in water contaminated by mine tailings. This ability was evidenced by a drop in Cr concentration from 3.1 ± 0.35 to 2.3 ± 0.74 mg/L mainly due to adsorption. Extracellular secretions are set up by the cell to prevent Cr from entering the cytoplasm. Samuel et al. (2021) indicated that biosorption depends on temperature, pH, biomass concentration, contact duration, and initial metal concentration. Majumder et al. (2017) showed that Cr^6+^ biosorption is endothermic and entropy-driven.

## 5 Conclusion

Adsorption of Cr by *Bacillus licheniformis, Bacillus megaterium, Byssochlamys* sp., and *Candida maltosa* strains isolated from tannery effluents was investigated in this study. Scanning electron microscopy combined with energy-dispersive X-ray spectroscopy revealed Cr characteristic peaks in cells treated with Cr^6+^. At 50 mg/L Cr^6+^, *Bacillus licheniformis* and *Candida maltosa* cells are rough. Extracellular secretions are reduced in *Bacillus megaterium*, and *Byssochlamys* sp. cells are tightly bound. Adsorption of Cr by all strains was better at 5 mg/L than at 50 mg/L. At 50 mg/L Cr^6+^ in LB medium, Cr wt.% of *Bacillus licheniformis, Bacillus megaterium, Byssochlamys* sp., and *Candida maltosa* are 0.29, 0.25, 0.35, and 0.23, respectively. FTIR analysis revealed metal-microbial interactions. It was found that phosphoryl terminals contribute to the Cr adsorption mechanism of *Bacillus licheniformis* while the amino acid ${\text{NH}}_{3}^{+}$ and ${\text{CO}}_{2}^{-}$ functional groups are involved in interaction of *Bacillus megaterium* strain with metal. Similarly, amino acid ${\text{NH}}_{3}^{+}$ infrared footprint show moderate changes in intensity and frequency, suggesting a possible contribution to the interaction between *Byssochlamys* sp. strains and metallic ions. The zeta potential analysis showed a variation in charges ranging from −10.63 ± 0.38 to −39.53 ± 1, −15.3 ± 2.18 to −30.4 ± 1.51, −8.98 ± 0.40 to −20.7 ± 0.98, and −10.15 ± 2.68 to −21.93 ± 2.08 for *Bacillus licheniformis, Bacillus megaterium, Byssochlamys* sp., and *Candida maltosa*. In addition, the zeta potential results indicated a Cr^6+^ adsorption limit of 100 mg/L for all strains. *Byssochlamys* sp. strain adsorb more Cr^6+^ at a concentration of 50 mg/L (0.35 wt.% Cr). It could therefore be used for the Cr bioremediation of tannery effluents. It is clear that in large-scale experiments, the adsorbed chromium can be recovered after washing the microbial cells, and the microbial biomass can act as a natural adsorbent. This green technology is highly advantageous.

## Data availability statement

The datasets presented in this study can be found in online repositories. The names of the repository/repositories and accession number(s) can be found in the article/supplementary material.

## Author contributions

AA: Data curation, Methodology, Writing—original draft, Writing—review & editing, Formal analysis. NR: Software, Formal analysis, Writing—original draft. MJ: Formal analysis, Writing—original draft. MH: Supervision, Investigation, Conceptualization, Writing—original draft, Writing—review & editing. YO: Methodology, Investigation, Formal analysis, Writing—original draft. LE: Supervision, Conceptualization, Methodology, Writing—original draft, Writing—review & editing.
